# The Effectiveness and Safety of Metformin Compared to Sulfonylureas in Diabetic Nephropathy: A Systematic Review

**DOI:** 10.7759/cureus.32286

**Published:** 2022-12-07

**Authors:** Chinmayi Sree Boddepalli, Sai Dheeraj Gutlapalli, Vamsi Krishna Lavu, Rana Abdelwahab Mohamed Abdelwahab, Ruimin Huang, Shanthi Potla, Sushen Bhalla, Yousif AlQabandi, Savitri Aninditha Nandula, Safeera Khan

**Affiliations:** 1 Internal Medicine, California Institute of Behavioral Neurosciences & Psychology, Fairfield, USA; 2 Dermatology, Mansoura University, Mansoura, EGY; 3 Dermatology, California Institute of Behavioral Neurosciences & Psychology, Fairfield, USA; 4 Psychiatry and Behavioral Sciences, California Institute of Behavioral Neurosciences & Psychology, Fairfield, USA; 5 Psychiatry, Avalon University School of Medicine, Cleveland, USA; 6 Ophthalmology, Al Bahar Ophthalmology Center, Sabah Area, KWT

**Keywords:** metmorfin, a systematic review, diabetes treatment, sulfonylurea, diabetic nephropathy (dn)

## Abstract

Metformin and sulphonylureas are the most commonly used first-line anti-diabetic agents. However, medical practice guidelines and clinical experience caution against using these drugs in severe diabetic kidney disease. Consequently, the choice of anti-diabetic medicine in various stages of diabetic nephropathy should balance the benefits and risks to the patient. We aim to synthesize available evidence on the effectiveness and safety of metformin concerning sulfonylureas in patients with diabetic renal disease. The COSMOS-E (Guidance on conducting systematic reviews and meta-analyses of observational studies of etiology) and MOOSE (Meta-Analyses and Systematic Reviews of Observational Studies in Epidemiology) guidelines were followed when designing the systematic review. The present study assessed the effectiveness of metformin and sulphonylurea monotherapy regarding renal function. Studies published from 2001 to 2022 were included. We have identified 570 records from PubMed, BioMed Central, LILACS (Literatura Latino-Americana e do Caribe em Ciências da Saúde), ScienceDirect, and PLoS (The Public Library of Science) Medicine databases. Eight cohort studies met the inclusion criteria. All studies reported adjusted hazard ratios with confidence limits. Metformin was found to be more effective in the following events: all-cause mortality, GFR (glomerular filtration rate), ESRD (end-stage renal disease) or death events, one-year risk of death or end-stage renal disease, cardiovascular events, heart failure hospitalization, and hypoglycemic episodes. However, metformin was less effective in acute renal replacement therapy, end-stage renal disease, and/or death, with a one-year risk of acute dialysis. Lactic acidosis was not significant with metformin. The present study recommends that metformin therapy is safe compared to sulfonylurea therapy in diabetic nephropathy patients, provided that the contraindications given in the guidelines are strictly adhered to.

## Introduction and background

Diabetes is one of the leading causes of kidney disease. According to the United States Renal Data System (USRDS) 2020 annual report, the share of diabetic patients among those who suffered from chronic kidney disease was about 24% [1]. The percentage of chronic kidney diseases among the diabetic population was about 33% [2]. The prevalence of macroalbuminuria is about 28% in the diabetic population, compared to a 5% prevalence in the non-diabetic and non-hypertensive population [3]. About 2% of patients without nephropathy diagnosed with type-2 diabetes developed microalbuminuria in a year. They transitioned to macroalbuminuria at an annual rate of 2.8% and elevated plasma creatinine or renal replacement therapy at a yearly rate of 2.3%. Most of these patients die before they progress into renal failure. Cardiovascular death is more common in diabetic nephropathy patients compared to diabetic patients with no nephropathy [4]. The above information indicates the high burden of diabetic nephropathy.

These patients are prescribed metformin and sulfonylureas as first-line anti-diabetic agents. Metformin has been in use since the 1950s in Europe and since the 1990s in the United States of America. Sulfonylureas have been available since 1956, and the second-generation sulfonylureas were introduced in 1984 [5].

Studies found that metformin and second-generation sulfonylureas were more effective in treating type 2 diabetes mellitus [6]. Even with the risk of hypoglycemia for sulfonylureas and gastrointestinal problems for metformin [7], few studies showed the risk of lactic acidosis associated with patients treated with metformin [8], and some studies found no such risk [7,9,10]. Metformin reduced the incidence of diabetes in high-risk individuals and proved to be a preventive medicine for diabetes [11,12,13]. Metformin was effective, even in diabetic individuals with chronic kidney disease, except in renal failure where eGFR (estimated glomerular filtration rate) was less than 30 ml/min/1.73 m2 [2,14,15]. The American Diabetes Association recommends metformin and comprehensive lifestyle management as first-line therapies for type 2 diabetes. If chronic kidney disease and heart failure predominate, sodium-glucose cotransporter-2 inhibitors and glucagon-like peptide-1 receptor agonists (GLP-1 RA) were recommended, as were basal insulin and sulfonylureas if A1c (glycated hemoglobin) exceeded the individual target level [16]. The American Association of Clinical Endocrinologists and American College of Endocrinology (AACE/ACE) Consensus Statement suggested sulfonylureas in the A1c range of 7.6% to 9.0% for greater glucose-lowering efficacy. As a result, metformin is the cornerstone of therapy because it is a safe insulin sensitizer, but it is also associated with a significant risk when combined with renal insufficiency.

According to the statement, sulfonylureas also have a moderate risk of renal insufficiency [17]. An examination of electronic primary care health records in the United Kingdom (United Kingdom) revealed an increase in the use of metformin as a first-line treatment for type 2 diabetes. Between 2000 and 2017, there was a decrease in the number of people starting sulfonylurea treatment. In patients with an eGFR of less than or equal to 30 mL/min/1.73 m2, sulfonylurea treatment has been undertaken [18]. Given the aforementioned guidelines, practices, and practical experiences, there has been some debate about the benefits and drawbacks of using metformin over sulfonylureas in diabetic nephropathy of varying severity. In this context, this systematic review aims to synthesize available evidence on the effectiveness and safety of metformin concerning sulfonylureas in patients with diabetic renal disease.

## Review

Methods

This systematic review was conducted and reported in adherence to COSMOS-E (Guidance on conducting systematic reviews and meta-analyses of observational studies of etiology) and MOOSE (Meta-analysis of Observational Studies in Epidemiology) guidelines [19,20]. The researchers developed this review proposal together. The search for studies, study selection, data extraction, and quality assessment were all done independently by the authors. Any disagreements were resolved by discussion.

Inclusion criteria: Patients diagnosed with type 2 diabetes mellitus for any duration, of any age or sex, and the geographical area where the population is of interest. Exposure: Use of metformin as monotherapy; the study measured the index date of exposure and reported the same to confirm the accuracy of the exposure measurement. Comparison: The use of any sulfonylurea of any dose as monotherapy. Besides, we included studies that investigated sulfonylureas as a comparative therapy and studies that analyzed the results between metformin and sulfonylurea separately. Outcome: The outcome of any adverse or protective health event was measured in terms of renal function, i.e., the outcome was measured in patients with kidney disease or in patients with and without kidney disease. There were no restrictions set for the type of studies. Studies published in the English language were included. Studies published in languages other than English were excluded from this review. Unpublished and gray literature was excluded.

Literature search

PubMed, BioMed Central, LILACS (Literatura Latino-Americana e do Caribe em Ciências da Saúde), ScienceDirect, and PLoS (Public Library of Science) Medicine databases were searched for recent studies published from 2001 to 2022. Searches were done between January 10 and February 8, 2022. The search strategies (given in Table 1) were piloted and refined in consensus with all authors. The references to relevant articles were also searched to identify additional eligible studies.

Study selection: The titles and abstracts of the identified publications were screened to 1. remove duplicates and eliminate studies that were not relevant to the review, and 2. remove studies that were not relevant to the review. The criteria used to filter the title and abstract were: research articles related to either of the following concepts: 1. diabetes and kidney (renal) diseases; 2. metformin; 3. sulfonylureas; and 4. any doubt about the research's exclusion from the review. Further full texts were screened and compared against the inclusion and exclusion criteria.

Data extraction: Data extraction sheets were developed to accommodate (a) bibliographic information (first author’s name, year of publication), (b) study design (type of study, duration of follow-up, number of participants in different groups, definitions of exposure and outcome), and (c) adjusted and unadjusted effect estimates with confidence intervals and p-values. The authors extracted the data independently.

Quality assessment: The Newcastle-Ottawa quality assessment checklist was used to measure the quality of the studies [21].

Statistical analysis: Included studies inconsistently reported adverse events. The definitions of adverse events varied across studies. Hence, we couldn’t conduct a meta-analysis.

Results

The databases and search strategies used for the present systematic review, along with the search results from each database, are shown in Table 1. Search strategies were built using words related to the health condition and interventions studied in the current review, balancing the sensitivity and specificity of the search.

**Table 1:** Databases and search strategies MeSH: Medical Subject Headings; LILACS: Literatura Latino-Americana e do Caribe em Ciências da Saúde; PLOS: Public Library of Science

Database	Search strategy	Results
PubMed	("nephropath*"[Title/Abstract] OR "glomerulonephritis"[MeSH Terms] OR "kidney diseases"[MeSH Terms]) AND 2001/01/01:2022/12/31[Date - Publication] AND (("diabetes mellitus"[MeSH Terms] OR "diabetes"[Title/Abstract]) AND 2001/01/01:2022/12/31[Date - Publication]) AND ("metformin"[MeSH Terms] AND 2001/01/01:2022/12/31[Date - Publication]) AND ("sulfonylurea compounds"[MeSH Terms] AND 2001/01/01:2022/12/31[Date - Publication])	58
BioMed Central	'(nephropathy* OR glomerulonephritis OR kidney OR renal) AND Diabetes AND Metformin AND sulfonylurea.'	467
LILACS	(((Nephropath* OR glomerulonephritis OR kidney OR renal) AND diabetes) AND metformin)	26
PLOS Medicine	(((abstract:nephropath*) OR abstract:glomerulonephritis) OR abstract:kidney) OR abstract:renal) AND abstract:diabetes) AND abstract:metformin	19

**Figure 1:**
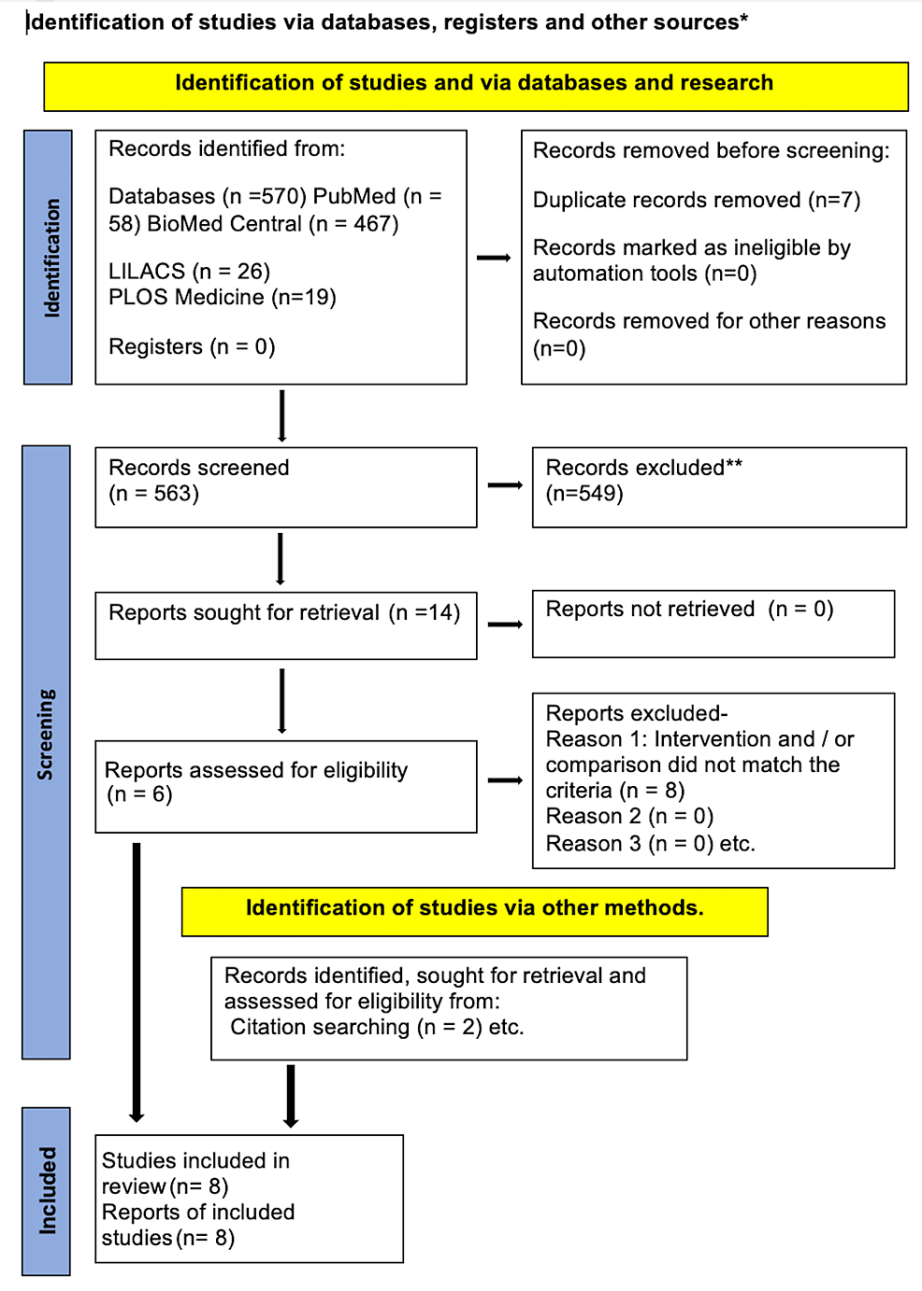
PRISMA (Preferred Reporting Items for Systematic Reviews and Meta-Analysis) 2020 Flow Diagram of Study Selection for the Review LILACS: Literatura Latino Americana em Ciências da Saúde; PLoS: Public Library of Science

A total of 570 records were identified after a database search, from which six studies were included in the review. Two more studies were added after a manual search of references. Details of the selection of research studies are in Figure 1 [22].

Study characteristics: All the studies were cohort studies. The characteristics of each study's population, exposures, and outcomes are given in Table 2. All the studies used data collected from population databases, registries, or repositories. Whitlock et al. [23] and Marcum ZA et al. [24] had no age restrictions, whereas Carlson et al. [25] excluded patients below 50 years. Richardson et al. [26], Chu PY et al. [27], and Marcum ZA et al. investigated outcomes in diabetic patients with kidney disease. Whitlock et al., Van Dalem J et al. [28], and Carlson N et al. studied outcomes in diabetic patients with known eGFR levels (normal and deteriorating kidney function). In two different cohort studies, Hung AM et al. studied the effects of anti-diabetic drugs on kidney function [29,30]. Patients with an eGFR of 30 ml/min/1.73 m2 were excluded by Carlson N et al. and Marcum ZA et al.Hung AM excluded patients with an eGFR <60ml/min/1.73m2 and eGFR >150ml/min/1.73m2.

**Table 2:** Characteristics of included studies CKD: chronic kidney disease; CPRD: clinical practice research datalink; eGFR=estimated glomerular filtration rate *remaining % are in other group/s; VHA: Veterans Health Administration; USA = United States of America; UK = United Kingdom; VISN 9: VA MidSouth Healthcare Network

First author (Year)-Country	Data source	Population	Participants= Number (%)	Study period	Index date
Richardson Jr. TL (2021), USA [25]	National VHA databases	≥ 18 years old, regular users of VHA care, with reduced kidney function	Metformin = 67762 (70) Sulfonylurea = 28979 (30)	2002-2016	Date of reaching a reduced kidney function threshold (eGFR of <60 mL/min per 1.73 m2 or serum creatinine level of ≥1.5 mg/dL for men or ≥1.4 mg/dL for women)
Patricia Y. Chu (2020), USA [26]	National VHA databases	≥ 18 years old, regular users of VHA care, and new users of metformin, glipizide, glyburide, or glimepiride.	Metformin = 67381 (70.06) Sulfonylurea = 28801 (29.94)	2002-2015	Date of reaching a reduced kidney function threshold
Reid H. Whitlock (2020), Canada [22]	The Repository at the Manitoba Centre for Health Policy	New users of metformin or sulfonylurea monotherapy with known eGFR	Metformin = 19990 (90.9) Sulfonylurea = 2006 (9.1)	2006-2017	On this date, an individual filled his or her first prescription for metformin or sulfonylurea.
Zachary A. Marcum (2017), USA [23]	National VHA databases at the Austin Information Technology Center	Patients with type 2 diabetes and CKD and new users of metformin or sulfonylurea monotherapy	Metformin = 111781 (63.77) Sulfonylurea = 63515 (36.23)	2003-2009	Date of 2nd prescription
Judith van Dalem (2016), UK [27]	CPRD	≥ 18 years old with at least one non-insulin antidiabetic prescription with eGFR≥ 60/30-59/<30	Metformin = 92005 (76.16), Sulfonylurea = 13224 (10.95)*	2004-2012	Date of the first prescription during data collection
Nicholas Carlson (2016), Denmark [24]	Danish National Registry	≥ 50 years old and initiating treatment with either metformin or sulphonylurea	Metformin = 119153 (70.74) Sulfonylurea = 49290 (29.26)	2000 - 2012	Treatment initiation date with no prior anti-diabetic medicine
Adriana M. Hung (2013), USA [28]	Mid-South VISN 9 Data Warehouse	≥ 18 years old with first oral anti-diabetic drug	Metformin = 7728 (58), Sulfonylurea = 4425 (33)	1999-2008	First oral hypoglycemic drug prescription was filled after at least 365 days of active use of VHA services without prescriptions filled for any hypoglycemic drug
Adriana M. Hung (2012), USA [29]	National VHA databases	≥ 18 years old with first oral anti-diabetic drug	Metformin = 61104 (65) Sulfonylurea = 30550 (33)	2001-2008	The first oral hypoglycemic drug prescription was filled after at least 365 days of active use of VHA services without prescriptions filled for any hypoglycemic drug

Quality assessment: All the included studies were validated using the Newcastle-Ottawa Quality Assessment Checklist for Cohort Studies [21]. All studies satisfied the quality standards for cohort studies. The details of the quality assessment are in Table 3. It was reported that individuals who did not have the required data or documents (up to about 30% of the study population in some studies) were excluded from the study.

**Table 3:** Validation of included studies using the Newcastle-Ottawa Quality Assessment Checklist for Cohort Studies Hb%: hemoglobin; HbA1c: hemoglobin A1c ACR: albumin to creatinine ratio; ARB: angiotensin II receptor blockers; BMI: body mass index; ACEI: angiotensin-converting enzyme inhibitors; eGFR: estimated glomerular filtration rate

		Selection				Comparability	Outcome				Quality of the study
		1	2	3	4	5	6	7	8	9	
		Representativeness of the exposed cohort	Selection of the non-exposed cohort	Ascertainment of exposure	Demonstration that the outcome of interest was not present at the start of the study	Comparability of cohorts based on the design or analysis controlled for confounders	Assessment of outcome	Was follow-up long enough for outcomes to occur	the median duration of follow-up and a brief rationale for the assessment above	Adequacy of follow-up among cohorts	
1	Reid H. Whitlock [23]	Somewhat representative (✓)	Drawn from the same community as the exposed cohort (✓)	Secure record (✓)	Yes (✓)	Study controls for age, sex, baseline lab results for Hb%, HbA1c, total cholesterol, urine ACR, and baseline medicines use (✓)	Record linkage (✓)	Yes (✓)	4.82 years. The report mentioned that the follow-up period was similar to that of other studies.	Complete follow-up; all subjects accounted for; retrospectively (✓)	Good quality
2	Adriana M. Hung [30]	Somewhat representative (✓)	Drawn from the same community as the exposed cohort (✓)	Secure record (✓)	Yes (✓)	Study controls (at analysis) for age (>/= 65 years or < 65 years), race, HbA1c(>7 or =7), and angiotensin-converting enzyme inhibitor, ARB, angiotensin receptor blocker ratio (✓)	Record linkage (✓)	Yes (✓)	Follow-up was measured in person-years until the study outcome or a censoring event occurred.	No statement	Good quality
3	Adriana M. Hung [29]	Somewhat representative (✓)	Drawn from the same community as the exposed cohort (✓)	Secure record (✓)	Yes (✓)	Study controls (at analysis) by adjusting for age, sex, race, baseline creatinine (fifth-degree polynomial), baseline blood pressure, history of hypertension, history of cardiovascular disease, baseline HbA1c, baseline BMI (third-degree polynomial), the use of ACEI or ARBs, diuretics, baseline number of medications (third-degree polynomial), year of cohort entry, number of outpatient visits, history of hospitalization at baseline, and marital status.	Record linkage (✓)	Yes (✓)	Follow-up was measured in person-years until the study outcome or a censoring event occurred.	No statement	Good quality
4	Patricia Y. Chu [27]	Somewhat representative (✓)	Drawn from the same community as the exposed cohort (✓)	Secure record (✓)	Yes (✓)	Study controls for mean-centered metformin dose, demographics, clinical information derived from the electronic health record, comorbidities, use of medications, and health care utilization are determined by adjusted models at the analysis stage (✓)	Record linkage (✓)	Can not say	1.2 years	Subjects who were not followed up on are unlikely to introduce bias; the descriptions of those who were not followed up on suggested no difference from those who were.(✓)	Good quality
5	Nicholas Carlson [25]	Somewhat representative (✓)	Drawn from the same community as the exposed cohort (✓)	Secure record (✓)	Yes (✓)	Study controls (at analysis) by adjusting results for gender, age, comorbidities, prescription medication, calendar time, and surgery (✓)	Record linkage (✓)	Can not say	One year	Subjects lost to follow-up are unlikely to introduce bias; the number lost is less than or equal to 20%. (✓)	Good quality
6	Zachary A. Marcum [24]	Somewhat representative (✓)	Drawn from the same community as the exposed cohort (✓)	Secure record (✓)	Yes (✓)	Study controls (at analysis) by adjusting results for demographic variables, health behaviors, the eGFR category, comorbid conditions, laboratory values, and cardiovascular medication use. (✓)	Record linkage (✓)	Yes (✓)	1.3 years. After this period, either the patients switched from monotherapy.	Complete follow-up; all subjects accounted for; retrospectively (✓)	Good quality
7	Judith van Dalem [28]	Somewhat representative (✓)	Drawn from the same community as the exposed cohort (✓)	Secure record (✓)	Yes (✓)	Study controls (at analysis) adjusted for age, sex, body mass index, alcohol use, smoking status, cardiovascular disease, chronic heart failure, and use of loop diuretics. (✓)	Record linkage (✓)	Yes (✓)	Mean 3.7 years, the follow-up period was pre-defined in the design	Complete follow-up; all subjects accounted for - retrospectively (✓)	Good quality
8	Richardson Jr TL [26]	Somewhat representative (✓)	Drawn from the same community as the exposed cohort (✓)	Secure record (✓)	Yes (✓)	Study controls (at analysis) are set by adjusting for age, sex, race, fiscal year, diabetes mellitus duration, and the Veterans Integrated Service Network of Care.	Record linkage (✓)	Yes (✓)	1.03, followed by an outcome (hospitalization for heart failure), a competing risk (drug non-persistence or death), or a censoring event (loss to follow-up or end of the study).	Complete follow-up; all subjects accounted for; retrospectively (✓)	Good quality
		Good quality: three or four stars in the selection domain, one or two stars in the comparability domain, and two or three stars in the outcome/exposure domain. Fair quality: two stars in the selection domain, one or two stars in the comparability domain, and two or three stars in the outcome/exposure domain. Poor quality: 0 or one star in the selection domain, 0 stars in the comparability domain, or 0 or one star in the outcome/exposure domain									

Outcomes: All included studies reported adjusted hazard ratios with confidence limits (HR (CI)). In addition, Whitlock et al. and Carlson et al. gave p-values. Carlson et al. and Marcum ZA et al. explained the outcomes in different eGFR groups. Hung AM et al. studied a persistent 25% or more decline in baseline eGFR as a GFR event. Whitlock et al. and Marcum ZA et al. studied the independent effect of eGFR on outcomes. Mortality was measured in terms of all-cause mortality or combined with renal disease events. The outcomes studied in the included studies are summarized in Table 4.

**Table 4:** Outcomes of the included studies Reference eGFR: 30-50; HR: hazard ratio; CI: confidence interval; GFR: glomerular filtration rate; eGFR=estimated glomerular filtration rate; ESRD: end stage renal disease

Outcomes	Participant subgroup	HR, (Cl) - P		Study
		Metformin	Sulfonylurea	
All-cause mortality	All participants	0.48 (0.40 - 0.58) - <0.001	Reference	Reid H. Whitlock [23]
	All participants	0.64 (0.60–0.68)	Reference	Zachary A. Marcum [24]
GFR, ESRD, or death events	All participants	Reference	1.20 (1.13 - 1.28)	Adriana M. Hung [30]
	All participants	0.82 (0.70, 0.97)	Reference	Adriana M. Hung [29]
Acute renal replacement therapy, end-stage renal disease, and/or death	All participants	1.53 (1.06 - 2.23)	Reference	Nicholas Carlson [25]
	eGFR (mL/min/1.63m²) >60	24.4 (-87.0 - 1107)	Reference	Nicholas Carlson [25]
	eGFR (mL/min/1.63m²) ≤60	366.2 (52.5 - 669.5)	Reference	Nicholas Carlson [25]
One-year risk of death or end-stage renal disease	All participants	0.66 (0.63 - 0.70) - <0.001	Reference	Nicholas Carlson [25]
	eGFR (mL/min/1.63m²) >60	0.55 (0.47 - 0.65) - <0.001	Reference	Nicholas Carlson [25]
	eGFR (mL/min/1.63m²) ≤60	0.70 (0.60 - 0.82) - <0.001	Reference	Nicholas Carlson [25]
One-year risk of acute dialysis	All participants	1.51 (1.06 - 2.17) - 0.022	Reference	Nicholas Carlson [25]
	eGFR (mL/min/1.63m²) >60	1.24 (0.48 - 3.24) - 0.658	Reference	Nicholas Carlson [25]
	eGFR (mL/min/1.63m²) ≤60	2.06 (1.12 - 3.71) - -.020	Reference	Nicholas Carlson [25]
GFR or ESRD events	All participants	Reference	1.20 (1.13 - 1.28)	Adriana M. Hung [30]
	All participants	0.85 (0.72, 1.01)	Reference	Adriana M. Hung [29]
Cardiovascular event	All participants	0.67 (0.52 - 0.86) - 0.002	Reference	Reid H. Whitlock [23]
Hospitalization with heart failure	All participants	0.85 (0.78–0.93)	Reference	Richardson Jr TL [26]
Hospitalization with lactic acidosis	All participants	1.21 (0.99, 1.48)	Reference	Patricia Y. Chu [27]
Hypoglycaemic episodes	All participants	0.14 (0.09 -0.20) - <0.001	Reference	Reid H. Whitlock [23]
	All participants	Reference	2.50 (2.23 - 2.82)	Judith van Dalem [28]
	eGFR (mL/min/1.63m²) >60	Reference	2.04 (1.73 - 2.41)	Nicholas Carlson [25]
	eGFR (mL/min/1.63m²) ≤60	Reference	5.20 (3.94 - 6.88) 4.96 (3.76 - 6.55)	Nicholas Carlson [25]

Discussion

Mortality in diabetic nephropathy: In the present review, mortality was reported in five studies. Mortality was reported in combination with four different groups of health conditions. Metformin users were at lower risk, except in the group where mortality was combined with "acute renal replacement therapy, end-stage renal disease, and/or death" (Table 4). Canadian research by Johnson JA et al. on new users of oral anti-diabetic agents suggested that all-cause mortality and cardiovascular deaths were lower in metformin monotherapy than in sulfonylureas [31].

Progression of renal diseases in diabetic nephropathy: The risk of progression of renal diseases was low in the metformin group, except the "one-year risk of acute dialysis" was high for metformin, though the p-value is not significant. (Table 4). The effect of metformin on delaying chronic kidney disease (CKD) progression is independent of its glucose control activity. Metformin reversed Gentamicin-induced renal dysfunction in a rat model, indicating that it can be renoprotective even in non-diabetic conditions [32].

Cardiovascular events in diabetic nephropathy: Cardiovascular events and hospitalizations for heart failure were less frequent in users of metformin monotherapy compared with sulfonylureas. (Table 4). Other studies in diabetic patients reported the cardiac advantages of metformin over sulfonylureas [33]. A Taiwan study reported the stroke-preventive effect of metformin [34]. In contrast, another case-control study in Taiwan on hemodialysis (CKD stage 5D) patients with type 2 diabetes identified a significantly higher risk of stroke in metformin users than in metformin nonusers [35].

Lactic acidosis in diabetic nephropathy: Chu YP reported that metformin was associated with lactic acidosis, but the confidence level was not significant concerning sulfonylurea monotherapy (HR (Cl) = 1.21 (0.99, 1.48)). Metformin therapy, according to a community-based cohort study conducted in the United States, is not associated with lactic acidosis in diabetics with an eGFR of 30 mL/min/1.73 m2 or higher [36].

Hypoglycemia in diabetic nephropathy: hypoglycemia was more frequently associated with the use of sulfonylurea and less common with metformin monotherapy (HR (Cl)-p = 0.14(0.09-0.20)-<0.001). 

Metformin is safe: According to European Union specifications, initiation of metformin was contraindicated in CKD with eGFR below 45 ml/min/1.73 m2, and sulfonylureas were contraindicated if eGFR was below 30 ml/min/1.73 m2 [37]. The present review identified that metformin monotherapy has a beneficial effect over sulfonylurea monotherapy in diabetic nephropathy patients if there are no contraindications. The pleiotropic effects of metformin may benefit cardiovascular protection, BMI (body mass index), BP (blood pressure), neuroprotection, nephroprotection, cancer, and PCOS (polycystic ovary syndrome) [38]. The use of metformin has been increased, either in monotherapy or in combinations [16].

A UK retrospective cohort study of 105123 diabetic patients found that sulfonylurea monotherapy was associated with a significant risk of all-cause mortality (adjusted hazard ratio (95% CI) = 1.749 (1.643-1.863), nephropathy (adjusted hazard ratio (95% CI) = 1.127 (1.011-1.265), and major adverse cardiac events (adjusted hazard ratio (95% CI) = 2.632 (2.198-3.1) Another retrospective study in the USA identified similar results. The study compared proteinuria (adjusted hazard ratio (95% CI) = 1.27 (0.93-1.74)) and eGFR reduction to <60 ml/min/1.73 m2 (adjusted hazard ratio (95% CI) = 1.41 (1.05-1.91)) in diabetics and reported that sulfonylurea exposure had a higher risk of developing these conditions compared to that of metformin [40]. A population-based study in England of 4,69,688 diabetic patients assessed that the risk of developing kidney failure was associated with sulfonylurea monotherapy when compared with metformin monotherapy (adjusted hazard ratio (95% CI) = 2.63 (2.25 to 3.06) [41].

Limitations

Metformin's reported association with lactic acidosis and contraindication for severe kidney disease may limit its use in diabetic nephropathy patients. Hence, patients with kidney disease may preferably start their treatment with sulfonylureas, according to these studies. This could be a confounder in determining the efficacy of metformin in diabetic nephropathy. The use of data from health registries and databases may not accurately provide the data required for the studies. Some of these retrospective cohort studies reported missing data.

## Conclusions

There have been few studies comparing the efficacy of metformin and sulfonylureas in patients with diabetic nephropathy. The evidence from observational studies is to be verified by RCTs (randomized controlled trials). The metformin group had a low risk of renal disease progression. Metformin also has cardiovascular advantages. Hypoglycemia was less common with metformin monotherapy. Metformin-associated mortality was low in diabetic nephropathy, but mortality was high in end-stage renal disease, compared to mortality associated with sulfonylureas. The present study recommends that metformin therapy is safe compared to sulfonylurea therapy in diabetic nephropathy patients, provided that the contraindications given in the guidelines are strictly adhered to.
